# High Fidelity System Modeling for High Quality Image Reconstruction in Clinical CT

**DOI:** 10.1371/journal.pone.0111625

**Published:** 2014-11-12

**Authors:** Synho Do, William Clem Karl, Sarabjeet Singh, Mannudeep Kalra, Tom Brady, Ellie Shin, Homer Pien

**Affiliations:** 1 Department of Radiology, Massachusetts General Hospital and Harvard Medical School, Boston, Massachusetts, United States of America; 2 Department of Electrical and Computer Engineering, Boston University, Boston, Massachusetts, United States of America; University of Navarra, Spain

## Abstract

Today, while many researchers focus on the improvement of the regularization term in IR algorithms, they pay less concern to the improvement of the fidelity term. In this paper, we hypothesize that improving the fidelity term will further improve IR image quality in low-dose scanning, which typically causes more noise. The purpose of this paper is to systematically test and examine the role of high-fidelity system models using raw data in the performance of iterative image reconstruction approach minimizing energy functional. We first isolated the fidelity term and analyzed the importance of using focal spot area modeling, flying focal spot location modeling, and active detector area modeling as opposed to just flying focal spot motion. We then compared images using different permutations of all three factors. Next, we tested the ability of the fidelity terms to retain signals upon application of the regularization term with all three factors. We then compared the differences between images generated by the proposed method and Filtered-Back-Projection. Lastly, we compared images of low-dose in vivo data using Filtered-Back-Projection, Iterative Reconstruction in Image Space, and the proposed method using raw data. The initial comparison of difference maps of images constructed showed that the focal spot area model and the active detector area model also have significant impacts on the quality of images produced. Upon application of the regularization term, images generated using all three factors were able to substantially decrease model mismatch error, artifacts, and noise. When the images generated by the proposed method were tested, conspicuity greatly increased, noise standard deviation decreased by 90% in homogeneous regions, and resolution also greatly improved. In conclusion, the improvement of the fidelity term to model clinical scanners is essential to generating higher quality images in low-dose imaging.

## Introduction

Computed tomography (CT) is one of the most commonly used diagnostic imaging modalities in modern medicine. CT enables rapid, non-invasive image acquisition at high resolutions. However, CT also exposes the patient to radiation [1], [2]. CT dosage can be decreased by lowering either the voltage or the flux. Lowering the voltage implies that the emitted photons are less energetic, reducing their ability to penetrate through the body. Lowering the flux reduces the number of photons emitted, further degrading the signal-to-noise ratio of the acquired data. Therefore, the consequence of low-dose CT imaging is that the resulting images are considerably noisier than images acquired with todays clinical doses [3].

The drive towards lower dose CT imaging (while maintaining the diagnostic quality of CT) has been an area of focus for the entire CT community [4]–[9]. Numerous approaches to dose reduction have been implemented in commercial systems including the use of filters [10]–[12], collimators [13], dose modulation [14], [15], prospective triggering [6], patient-specific protocols [16], [17], and more [12], [18]. One additional component to the current repertoire of low-dose CT scanning techniques is the use of new image reconstruction techniques.

Through recent studies, iterative reconstruction (IR) algorithms have been shown to be more robust than FBP algorithms in regards to the presence of noise and artifacts [8], [19]–[25]. Numerous researchers have discussed different aspects of formulations [26]–[31] and optimization approaches [32]–[36]. However, we have found that having a high fidelity model of the imaging system is also a critical factor in the reconstruction of high quality images; this is an aspect of iterative reconstruction algorithms which has often been either neglected or substantially simplified [37], [38].

A critical component of tomographic IR algorithms is the accuracy of the forward system model. In positron emission tomography (PET), the forward system model consists of a geometric projection matrix and a sinogram blurring matrix, which can be either measured or simulated [39], [40]. It is shown that the combined model improves resolution and contrast-to-noise ratio in PET imaging [41]. It is also possible to reuse the stored system matrix to improve computation time because the PET scanner is stationary, making it relatively easy to factorize the system model based on symmetric geometry. A similar method is applied to single photon emission computed tomography (SPECT) for the estimation of the depth-dependent component of the point spread function (PSF) [42]. However, it is a challenging task to derive an explicit system matrix in clinical CT for the following reasons: i) each scan has a different scan length and pitch based on the scanning protocol, and ii) it is very hard to find symmetries in cone beam helical CT scans because the source-detector set has a functional misalignment (i.e., a quarter of a detector offset [43]) and view-by-view deflections of the X-ray source spot (i.e., flying focal spot (FFS) [44]).

In this paper, we show the systematic implementation of accurate system modeling for an IRT in clinical CT. A similar approach for PET [45] was derived from an analytical formula for calculating error propagation in a reconstructed image from the system matrix. In addition, in the cone-beam CT, the beam divergence and the rotation of the X-ray source and detector unit give space-variant effect on image. Since we do not use a system matrix as in PET, we integrate all the functional misalignment and fabrication limitations with on-the-fly calculation method so that the space-invariant nature is embedded in the forward model. Therefore, when we run image reconstruction algorithm, we set up on/off parameters for each modular model. That is one of major differences of our results compared to the previous 2D or phantom simulation works.

Also, there are algorithms (ASIR, IRIS, iDose, VEO, etc.) implemented in clinical scanners by vendors, but the technical description and detailed methods are not available to the research community. In this paper, we systematically demonstrate the necessity of implementing focal spot area, flying focal spot, and detector area in the forward system model to generate higher quality images. We also compare our raw-data-domain IRT with a mathematical formulation of image domain iteration called Iterative Reconstruction in Image Space (IRIS). The purpose of this paper is to examine the role of high-fidelity system models in the performance of the iterative image reconstruction approach minimizing energy functional. This paper is organized as follows. In section 2, we mathematically describe iterative image reconstruction and the components of proposed system models. In section 3, we present some initial results on phantom and in vivo data. In section 4, we summarize our findings and conclusions.

## Methods

In this section, we describe the mathematical formulation of IRT and a detailed forward system modeling method. The forward system modeling method can be decomposed into a series of components to increase modeling accuracy. We structure a three-component model that incorporates the most important elements of the system model. Each component can be replaced by a specific scanner parameter or vendor-specific model. The accuracy of this system model is critical in the improvement of image quality of a reconstructed image.

### On-the-fly System Modeling and Reconstruction Formulation for Clinical Scanner

We assume a transmission CT system with a field of x-ray attenuation coefficients 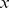 and projection operator 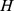 as modeled by:
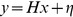
(1)where 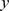 denotes the projection sinogram, and 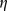 denotes noise. We formulate our reconstruction problem by the following equation:
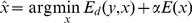
(2)where 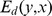 is the data fidelity term between image 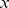 and sinogram 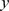 via the projection process. The second term 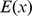 is the prior, or regularization term, and 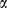 is the weighting term. We formulate the fidelity term as:
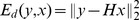
(3)where 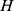 is the system matrix, or projection process. One example of the regularization term 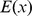 is the 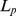 norm:
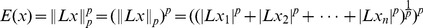
(4)


When 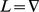 and 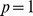, 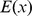 becomes a Total Variation (TV) regularizer, which is commonly used to suppress noise and preserve edges in the image [46], [47]. From a modeling perspective, we make the assumption that 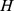, the system matrix, can be decomposed into a series of component models:
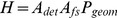
(5)


The models include a geometric projector (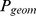), a focal spot model (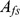), and an active detector response function (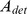). By decomposing a system matrix 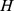 into sub-components, the implementation of complex clinical scanner modeling becomes more feasible. This approach also increases the usability of a single developed code across multiple CT systems, as opposed to requiring entirely different projectors for each system.

In this paper, we used the least-squares (LS) solution without the regularization term and TV solution in Equation (2) and (4) for comparison. The lagged diffusivity fixed-point method [46], [48], where we iteratively approximated the cost by a weighted quadratic cost and then solved the resulting linear normal equations using pre-conditioned conjugated gradient (CG) iterations, is used to minimize the energy functional in Equation (2) [49].

### Focal Spot Area Modeling

**Figure 1:**
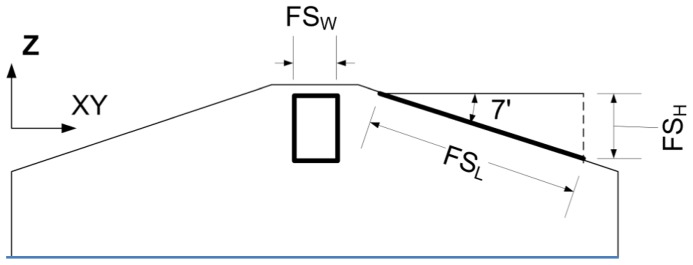
Focal spot area diagram: The length (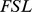), width (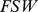), and height (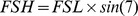) of the focal spot area (rectangular shape) are denoted in the diagram.

A focal spot is the region where electrons transfer their energy to target atoms in order to generate X-rays. In many cases, the focal spot is approximated as a point model, but in reality, the focal spot consists of a finite area (i.e., 0.3 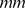 to 0.8 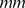) [50]. Furthermore, the size of this area changes with scanner settings (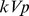 or 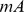), an important consideration in regards to image reconstruction of low dose scans. Figure 1 illustrates the focal spot area with the length (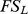), width (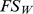), and height (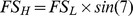) of the area in the diagram. This sub-module should be included for accurate forward system modeling.

### Flying Focal Spot (FFS) Modeling

**Figure 2:**
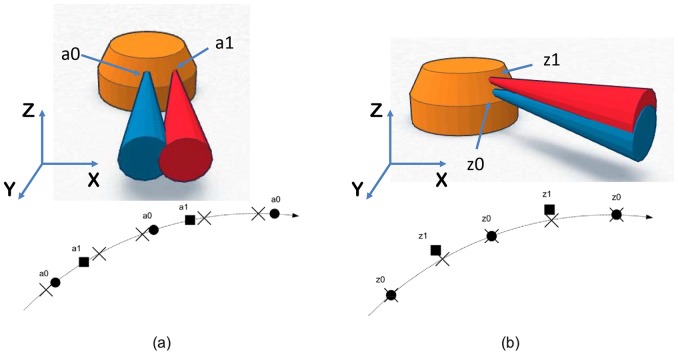
Flying Focal Spot (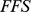) modeling: (a) 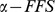 model shows deflected 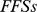 to the angular direction and (b) 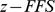 model shows 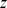-directional deflections.

**Figure 3:**
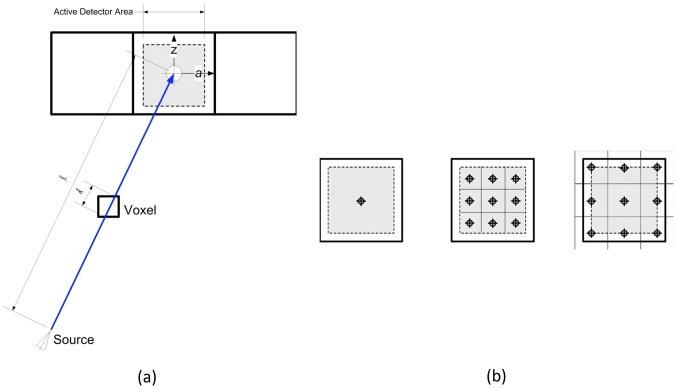
Diagram of active area of detector element: (a) Siddon-type ray-based projector calculates the ratio of intersections of the ray (i.e., 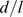), (b) Gray area is the active region of single detector element, Left: single element model, middle: multiple elements by limiting active area of detector, right: multiple elements by assigning rays to the boundary of active area of detector element.

The detector elements form an equiangular concentric cylindrical structure with 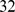 rows and 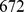 channels (i.e., 1st generation Dual Source CT, Siemens, Definition) with FFS models as shown in Figure 2. We assume the active area of all detector elements (i.e., 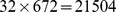) is identical for all elements according to manufacturer specifications. In Siddon-type ray-based projectors, a single ray sum is calculated for a single detector element by using the ratio of intersections of the ray with equally spaced parallel lines [51]. For our IR technique, we calculate a bundle of rays to simulate the virtual ray, which shapes the volume from the focal spot area to the active detector area. The ratio of the active detector area to physical spacing between detector elements was provided to us by the scanner manufacturer as 85% in the angular direction and 80% in the z-direction as shown in Figure 3. These ratios can be changed for different systems.

The Siddon projector calculates only the weighted sums of the portion of the ray that intersects through each voxel without considering and compensating for the neighboring voxels, generating aliasing artifacts [52]. Multiple rays in the volume beam can be used to compensate for this aliasing effect at the expense of over-sampling the image grid [53]. We have additionally implemented a version of the Siddon projector which does not require recursion [54], thus making it amendable to parallel implementations [55].

Figure 3-(b) shows how we divide active sub-elements to compute ray-sums. In Figure 3-(b), a single element model, as well as a multiple element model that strictly limits the active area of the detector (i.e., middle sub-figure), is depicted. We have noticed, however, that applications with reconstructions on voxel sizes that are finer than the detector size itself requires a greater over-sampling of the detector. In this case, not only does the computational demand increase, but the gap between the active areas of two adjacent detectors begin to introduce artifacts. As such, we have implemented the active area model depicted in the right sub-figure of Figure 3-(b), where the rays intersect the major boundary points, leading to a higher quality reconstruction.

## Results

We show two experimental results in this section. For the phantom study, we focus on the comparison between the effects of each model on the LS images with a cone beam phantom (QRM, Moehrendorf, Germany) with respect to conspicuity improvement, noise statistics, and resolution. In an in vivo study, we show clinical evidence that supports the proposed approach with subjective assessment. The proposed method is compared with conventional FBP and image domain IR (IRIS) algorithms in a low dose scan. In this case, we used the same raw data for image reconstructions.

### Phantom Study

A cone beam phantom with a spatial resolution section with 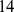 circularly aligned line-patterns varying from 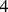 to 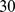 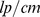 was scanned on a dual source 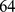-slice multi-detector row CT (Definition, Siemens Healthcare, Forchheim, Germany) using the following parameters: detector collimation  = 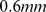, table speed 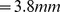 per gantry rotation, gantry rotation 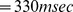, tube current 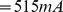, and tube voltage 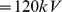.

In the following sections we demonstrate the impact of our various system modules. We will use the following notation: the triplet 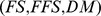 to denote with a 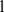 or 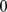, whether the focal spot model, flying focal spot model, and detector model, respectively, are turned on (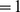) or off (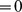). Thus, for example, 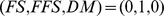 indicates that the flying focal spot model is turned on, while the other two system models are turned off.

**Figure 4:**
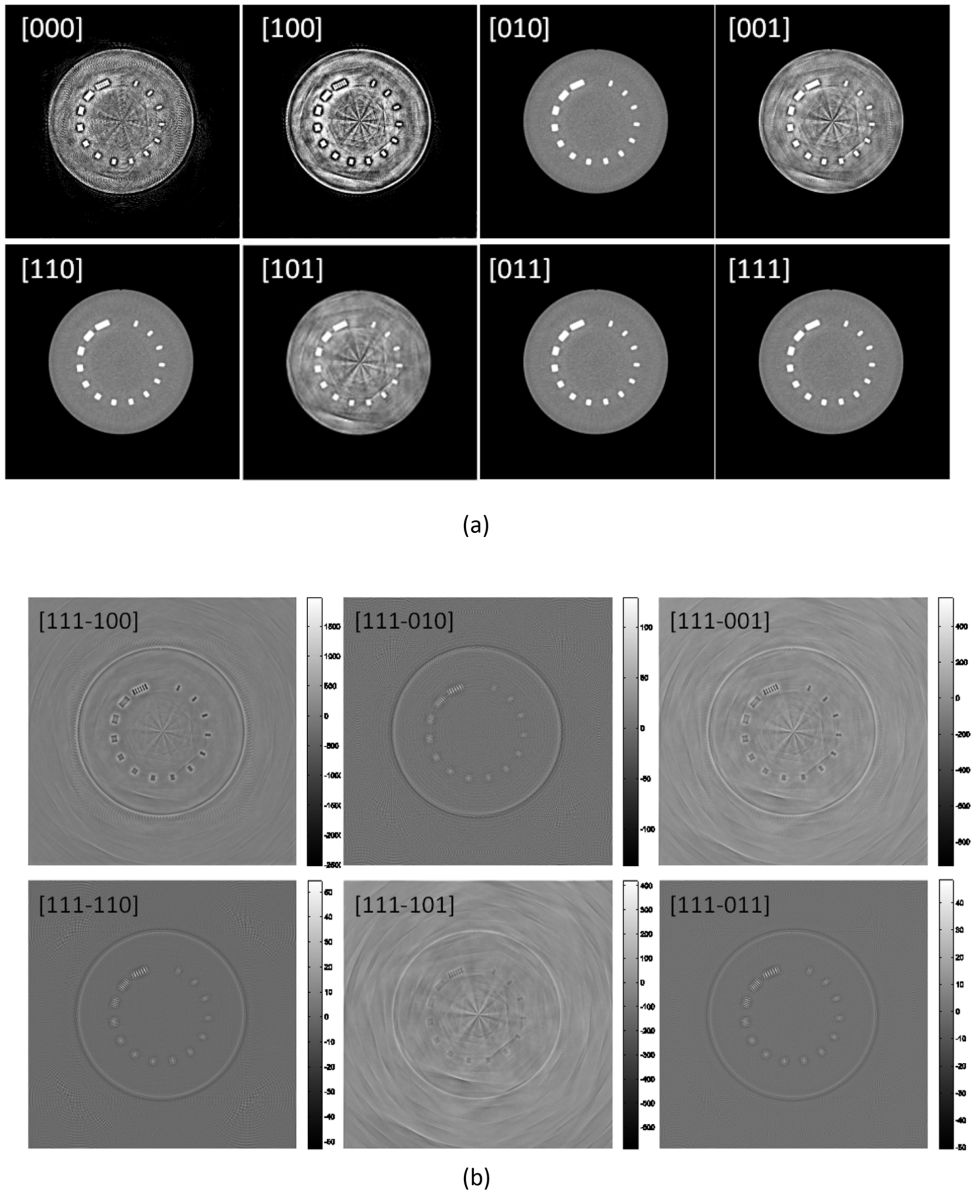
Modular system model effects: (a) Images are displayed in 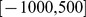 HU and (b) difference maps are displayed in dynamic contrast range. 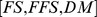
: 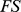: Focal Spot model, 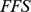: Flying Focal Spot model, and 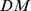: Detector model.

Figure 4 shows the LS solutions reconstructed using all permutations of 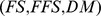. Additionally, the differences between these permutations and the case in which all models are turned on (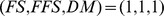) are shown. All reconstructed images are shown with a windowing level of 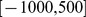, and difference images are shown with the full dynamic range of each difference so that patterns of artifacts are visible. The model without FFS generates the stellar shape artifact from the center of the rotation and it causes major deterioration of image quality. Therefore, FFS modeling is one of the most important components of clinical system modeling. The image quality evaluations in analytic reconstruction methods are shown in papers [44], [56]. In analytic reconstruction methods, the locations of X-ray source and detector elements are the only models that can be implemented in the algorithm, so it is easy to overlook the importance of FS and DM.

In Figure 4, we can visually compare image qualities of Siddon-type model with FFS 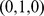 and the proposed method 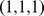 including focal spot and detector models to acquire a more accurate system model and to remove Moire patterns. There are only small differences between the two models, especially around the edges of the image, but eventually these will cause a significant change in the final image (i.e., TV regularization), especially in low dose scans. To suppress noise in low dose imaging, we frequently use regularization terms in Equation (2) with which we suppress noise by keeping the structure components of the image. When there are small model discrepancies related to the fidelity term in Equation (3), the mismatches can be concealed by noise and may cause resolution degradation and eventually poor contrast.

**Figure 5:**
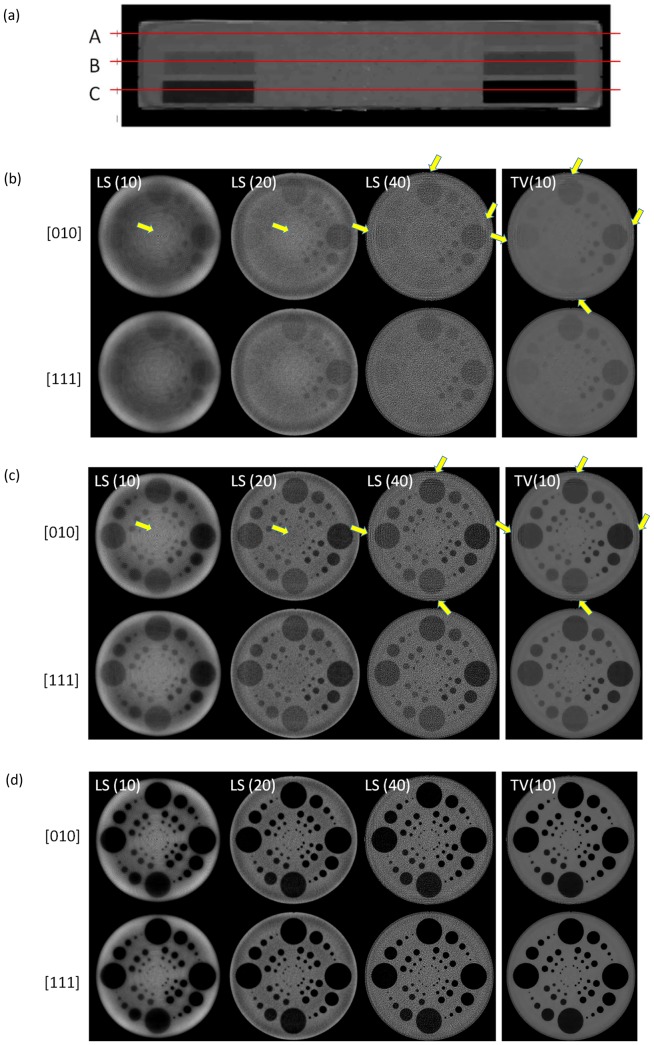
A modeling effect comparison on LS and TV images: (a) Coronal view of soft contrast section of phantom, (b), (c), and (d) show axial views of line A, B, and C respectively. The FFS only model (so called Siddon Model, 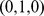) shows circular line artifacts in LS and TV as well. In contrast, the proposed model (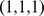) shows high quality image even in LS without regularization term and significant noise suppression effect on TV.

To compare artifact propagation, we compare the LS and TV images with a soft contrast section of the phantom. The three cross-sections of the soft contrast region are displayed in Figure 5-(a). Figure 5-(b), (c), and (d) show the axial views of line A, B, and C respectively. The top images are from Siddon-type model with 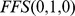 and the bottom images are from the proposed method 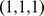. By the number of iteration (i.e., LS: 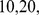 and 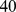 iteration, TV: 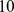 iteration), we can observe circular line artifacts on LS and TV, as well as on the Siddon-type model. It is especially more obvious in a very low contrast case (Figure 5-(b)). However, the images reconstructed by the proposed method show high quality images without any artifacts in both LS and TV. As expected, the TV images show significant noise suppression.

To observe the effects of iterations, we simulated a Siddon-type projector with proper 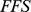 model 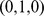 without 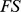 and 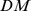, and a complete model 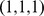 including all modular models in Section 2. Both methods used the same optimization algorithm and code (C++ and Open MPI) to reconstruct images and consecutively calculate the NRMSE (Normalized Root Mean Square Error):
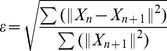
(6)where 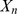 is the 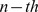 iteration result of LS-solution with 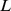 elements.

**Figure 6:**
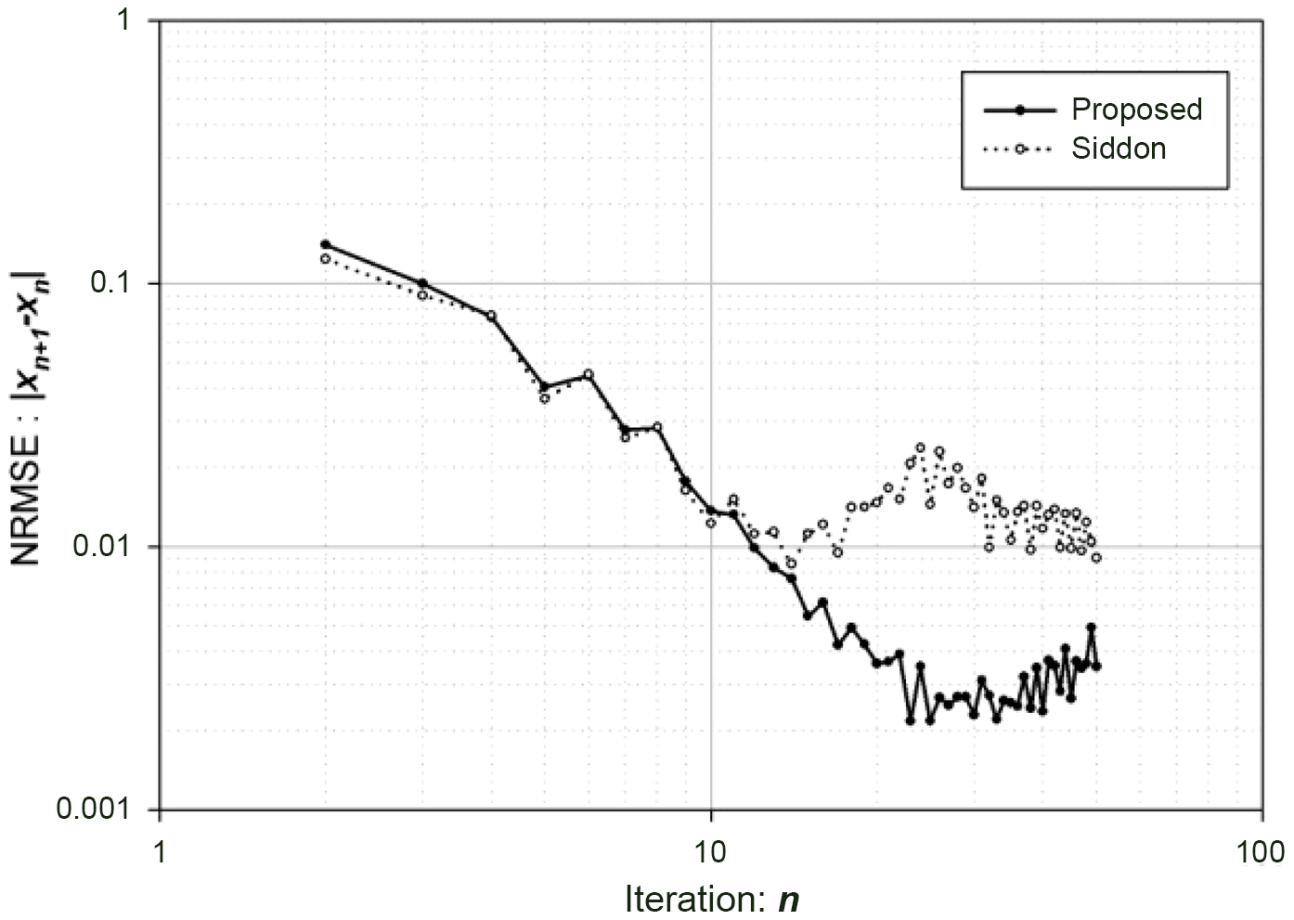
Consecutive error plot of Siddon 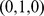 and the proposed method 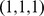. The NRMSEs of two consecutive images are calculated and displayed in log-log plot. The proposed method exhibits smaller consecutive errors after 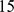 iterations compared to Siddon method and reaches smaller modeling error.

Figure 6 compares the consecutive errors with iteration for Siddon 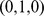 and the proposed method 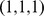 in the log-log scale. The Siddon-type projector shows similar updates to the proposed method until iteration-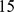, where it plateaus and then fluctuates. The resulting image from the Siddon projector does not provide the best image even though it reaches the solution of Equation (3). In contrast, the NRMSEs for our proposed method become smaller even after 15-iterations because the modeling error decreases with increasing iterations. However, the high frequency and noise components of the 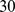 iterations become dominant in the system modeling.

Note that the proposed method exhibits significant modeling error reduction in terms of visual evaluation and NRMSE taking into account the nonlinear scaling of log-log plot.

#### Conspicuity Improvements

**Figure 7:**
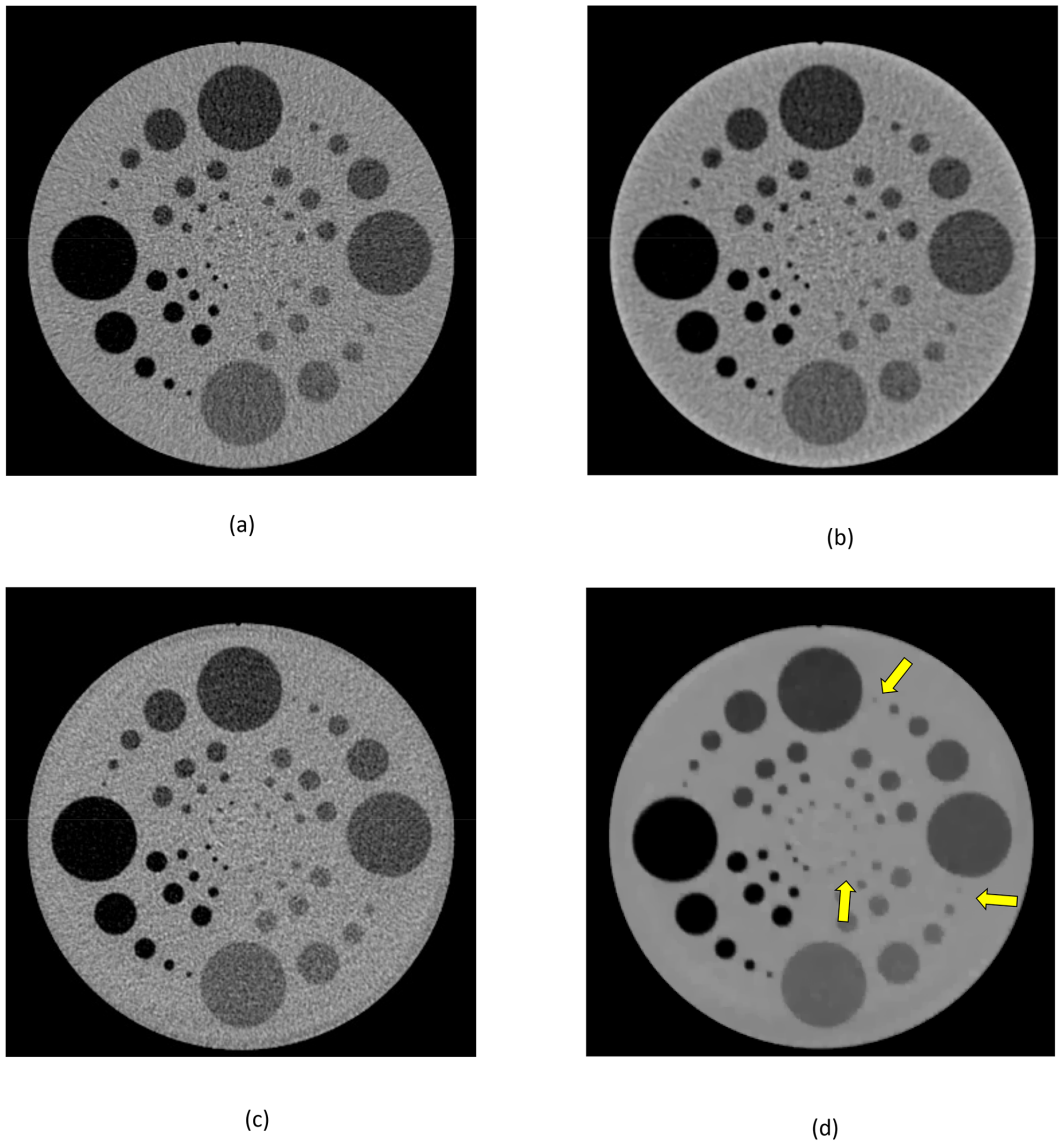
Soft contrast and conspicuity comparison for (a) FBP with sharp kernel filtering, (b) FBP with soft kernel filtering, (c) Least-Squares solution after 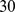-iteration, and (d) Total Variation (TV) image after 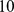-iteration with (c) initialization 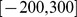.

In Figure 7, we display a contrast resolution section of QRM phantoms scanned in the clinical system. We show four groups of circles with different attenuation coefficients in Hounsfield Units (HU) (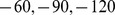, and 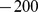 HU) in tissue equivalent background (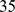 HU) at 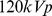. Each group consists of 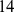 circular inserts with different diameters (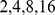, and 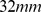). We used the same raw data for the comparison of four different reconstruction methods: Figure 7-(a) FBP with sharp kernel filtering, (b) FBP with soft kernel filtering, (c) Least-Squares (LS) solution after 30-iterations, and (d) Total Variation (TV) image after 10-iterations with (c) initialization.

We found that it is difficult to detect low contrast circles (for example, 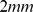 circles in 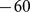 and 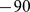 HU groups) visually as shown in Figure 7-(a), (b), and (c). Notice that there is no improvement of conspicuity in small circles with low contrast even with the smoothing kernel, Figure 7-(b). Basically, it suppresses high frequency noise components on the image without keeping small and low contrast information, which is key to the evaluation of low contrast tissues and lesions in most soft tissues such as the brain, liver, spleen, and lymph nodes, given subtle or low differences in HU values between organs and several of these legions (i.e., neoplasms and infarcts).

However, we can easily identify 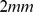 circles in 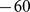 and 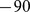 HU groups from Figure 7-(d). The TV image can be initialized on FBP image and can replace the LS solution.

#### Noise Statistics

**Figure 8:**
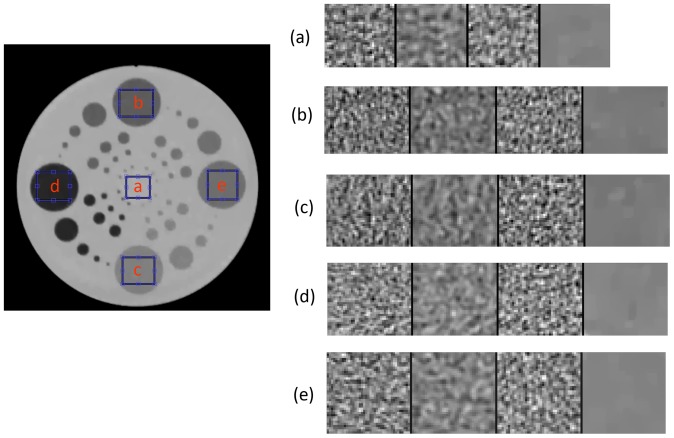
Noise patches comparison for the four images in **Figure 8**. Each sampled region is concatenated with dividing columns (zeros) and displayed in dynamic contrast window to show noticeable differences. From left to right, FBP with sharp kernel filtering, FBP with soft kernel filtering, IRT (LS), and IRT (TV).

Figure 8 compares noise patches from five noise regions for each image in Figure 7: one from each tissue equivalent region at the center of the Phantom and four 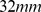 circles from different HU groups (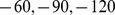, and 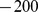 HU). Each sampled region is concatenated with dividing columns (zeros) and displayed in a dynamic contrast window to show noticeable differences. The FBP with sharp kernel filtering and LS solutions show similar noise patterns. As shown in Table 1, a smoother and lower spatial frequency kernel FBP with soft kernel filtering has lower noise compared to that of a sharper and higher spatial frequency kernel FBP (sharp kernel). However, TV shows a strong noise suppression capability and retains visibility of small and low-contrast circular objects that are, in our opinion, from accurate system modeling.

**Table 1:** Shows the measurements of noise mean (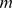) and standard deviation (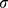) for each patch from four difference reconstruction images.

		FBP(sharp kernel)	FBP(soft kernel)	IRT(LS)	IRT (TV)
Patch a	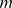	35.41	35.67	43.09	40.82
	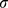	28.39	17.78	26.60	2.31
Patch b	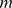	−83.25	−84.19	−78.41	−78.92
	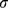	21.81	13.14	22.35	2.04
Patch c	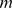	−26.99	−27.02	−24.23	−24.58
	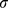	20.25	12.09	23.11	2.05
Patch d	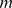	−163.35	−164.98	−163.40	−163.64
	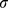	19.07	11.66	21.90	2.29
Patch e	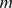	−54.57	−54.60	−51.37	−52.48
	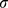	21.04	12.40	21.60	1.28

FBP (soft kernel) has smaller standard deviation than FBP (sharp kernel) and IRT (LS) but the IRT (TV) shows the smallest noise standard deviation.

The image reconstruction formulation in Equation (2) emphasizes that the energy functional aggregates a fidelity term and a regularization term. When the system model is not accurate enough to model details of the system, the TV-regularization (Equation (4)) of the energy functional (Equation (2)) smears or even loses the signal components associated with lower HU rather than noise when enforcing the smoothness constraint (i.e., 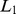-norm) as in Equation (4). This can even occur to greater signal components in low dose CT data, when the system model is inaccurate and iteration proceeds to suppress amplified noise.

On the other hand, the accurate system model sustains small signal components in the fidelity term so that it eventually reveals hidden signal components under the noise components.

#### Resolution

The reconstruction parameters of FBP and the proposed IRT method 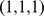 are set to be the same as slice-thickness 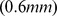.

**Figure 9:**
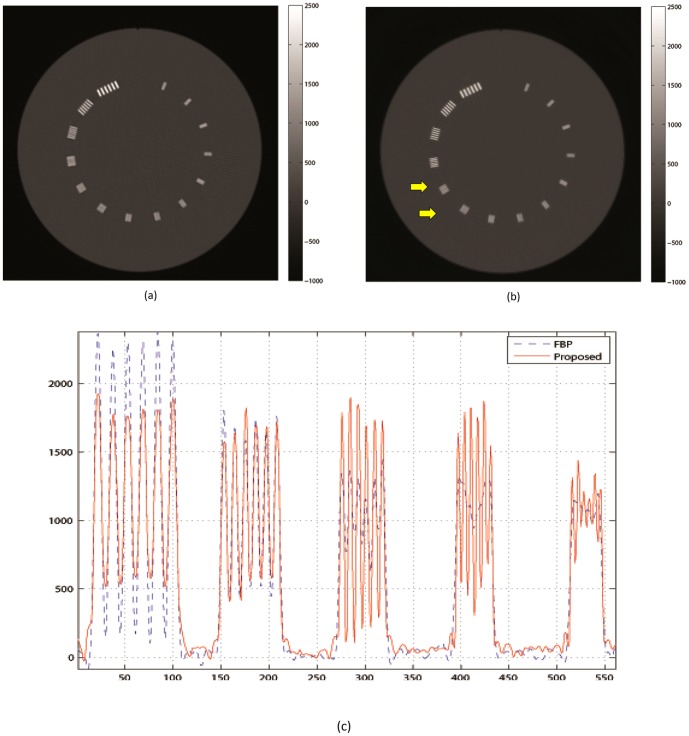
Spatial resolution bar pattern comparison: (a) FBP (sharp kernel) and (b) TV with high fidelity term image with proposed model (111) are displayed in 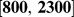 HU. (a) shows clear separations of 4, 6, and 8 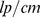 and (b) presents improved resolution showing 10 and 12 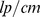 bar patterns. (c) compares profiles of spatial resolution inserts.

The spatial resolution bar patterns are displayed in Figure 9 with a 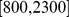 contrast window. FBP (sharp kernel) and FBP (soft kernel) had similar resolutions, so only the better FBP (sharp kernel) is displayed in Figure 9-(a) and compared to the TV with advanced model in Figure 9-(b). Note that the arrows in Figure 9-(b) indicate 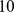 and 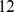 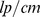 inserts, which points to clear improvement of spatial resolution on the TV with advanced modeling image.

### In vivo study

In the in vivo study, we only show clinical evidences of the proposed approach with subjective assessment. The proposed method is compared with conventional FBP and image domain IR algorithm (IRIS) in a low dose scan. In this case, we used the same raw data for image reconstructions. This study was conducted in compliance with the Health Insurance Portability and Accountability Act (HIPAA) and used a scan protocol approved by the Massachusetts General Hospital Institutional Review Board (IRB). We obtained written informed consent as per Federal U.S. guidelines. All procedures in this study were performed in accordance with the approved protocol.

A patient was scanned on a dual source 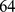-slice MDCT (1st generation DSCT Definition, Siemens Medical Solutions) using routine abdominal CT protocols. The scan parameters were 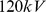, 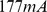, and 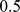 second gantry rotation. The reconstructed images show low dose image quality by reconstructing images with only half the data (i.e., detector A).

For this case, images with the volume 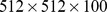 were reconstructed. For FBP, the sharp kernel on the scanner was utilized. For Iterative Reconstruction in Image Space (IRIS) [57], [58], the corresponding sharp kernel was chosen. IRIS is developed on a novel mathematical algorithm through iterative formation. The image domain iteration is initiated after it reconstructs a master volume, which is reconstructed based on how the scan projections provide image detail information, while reducing noise and enhancing object contrast step by step. IRIS utilizes well-established convolution kernels so that it is very fast compared to raw data domain iterative reconstruction.

In this experiment, we compare three different reconstruction approaches: the conventional FBP method, image domain iterative IRIS, and raw data domain IRT with the proposed methods. We have used 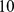-iterations with 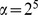 for IRT reconstruction with the proposed system modeling method.

To compare images, we defined a priori the regions of comparison. For SNR, the signal is defined over the region containing the hepatic artery, while the background standard deviation is chosen from patches (over 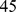 slices) of the liver without vasculature. We define CNR as:
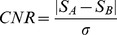
(7)where 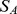 and 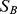 are mean signal intensities of signal producing structures of the liver (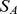) and the mean signal of the camper's fascia (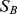), respectively. To obtain CNR background statistics, the standard deviation of the camper's fascia over 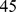 slices was computed, and is denoted by 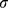 by in Equation (7).

**Figure 10:**
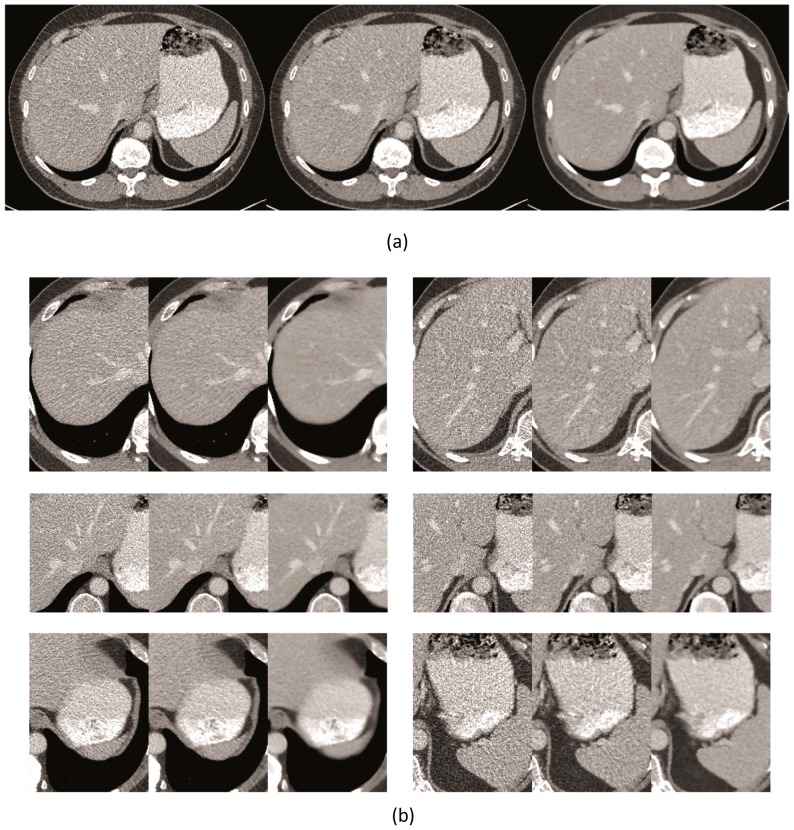
Comparison of reconstruction methods on half-dose images: (a) Reconstructed images, with (L) FBP, (M) IRIS, and (R) TV with advanced system modeling, (b) Zoomed images from different slices; each sub-figure shows (L) FBP, (M) IRIS, and (R) TV with advanced system modeling. Display in 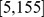 HU.

A comparison of reconstruction algorithms for the half-dose scan is shown in Figure 10. Although the added noise associated with this low-dose scan is apparent, no undesired texturing appears in this set of images either. The proposed method shows better visual impression compared to FBP and IRIS.

We also tabulate the SNR and CNR of the low-dose scans, in Table 2 and 3, respectively. The proposed method preserves the signal/contrast at much reduced noise for the low-dose acquisition. In this paper, we choose the regularization parameter based on clinician's feedback so it can be improved by processing more cases with broad feedback from multiple radiologists.

**Table 2:** SNR comparison of the three reconstruction methods for the half-dose dataset in Figure 10.

	FBP	IRIS	TV with high fidelity term
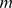	173.43	169.78	169.60
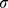	43.66	20.99	13.17
SNR ( 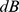 )	27.59	41.81	51.11

**Table 3:** CNR comparison of the three reconstruction methods for the half-dose dataset in Figure 10.

	FBP	IRIS	TV with high fidelity term
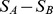	192.24	192.40	198.52
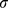	35.78	18.55	6.88
CNR	5.37	10.37	28.84

In this study, we showed the efficacy and impact of the proposed method in the real clinical scanner.

## Discussions and Conclusions

Modern CT systems are highly complex, and different reconstruction algorithms go to various lengths to model such complexities. In this paper, we show that the accurate modeling of system components such as focal spot area, flying focal spot, and active detector area can make a significant difference in the quality of reconstructed images.

Our phantom and patient studies show that the proposed technique can improve image quality (low contrast, noise statistics, spatial resolution, and visual impression). We have introduced a modular system modeling framework for a sophisticated clinical CT scanner. Even within the same system, some functions can be turned off or manipulated for clinical purposes. The advanced functions of state-of-art CT scanners need to be modeled accordingly for high quality image reconstruction. These parameter changes are meticulously recorded in the header files of raw data. None of these functions can be ignored for accurate system modeling to develop high-fidelity characteristics of an iterative algorithm.

As shown in Figure 4, there are sub-modules of the system that cause a small mismatch in system modeling, but these can be propagated through iterations, making them very hard to correct or compensate for by post-processing or utilization of regularization terms. Many studies claim that they can produce high quality SNR images with simple phantoms (having a few high density structures with homogeneous background) in low dose imaging; however, it is very hard to contain the small low-contrast structure in the final results without advanced system modeling. Without satisfying the fidelity term of the energy functional in Equation (3), we cannot guarantee that the reconstructed image is “the only stable solution” of this ill-posed image reconstruction problem.

At 50% dose, both IRIS and the proposed TV with advanced system modeling were found to be diagnostically acceptable. Although the proposed TV provided objectively superior images in terms of SNR and CNR, this image quality was achieved through a significant amount of processing. As a technique that achieves fast computations while maintaining good image quality, a hybrid method (such as IRIS) may potentially be a promising approach.

Future work will also address a variety of dose reductions on cadavers, and we anticipate being able to reduce computation time for the proposed advanced system modeling by implementing it on parallel computing architectures.
